# Sensing Classroom Temperature, Relative Humidity, Illuminance, CO_2_, and Noise: An Integral Solution Based on an IoT Device for Dense Deployments

**DOI:** 10.3390/s24165129

**Published:** 2024-08-08

**Authors:** Wilmar Hernandez, Norberto Cañas

**Affiliations:** 1 Carrera de Ingenieria Electronica y Automatizacion, Facultad de Ingenieria y Ciencias Aplicadas, Universidad de las Americas, Quito 170513, Ecuador; 2 Escuela Técnica Superior de Ingeniería de Sistemas Informáticos, Universidad Politécnica de Madrid, 28031 Madrid, Spain; norberto.canas@upm.es

**Keywords:** remote sensors, sensor networks, sensor applications in the environment

## Abstract

Maintaining optimal Indoor Environmental Quality (IEQ) requires continuous measurement of certain variables. To this end, ASHRAE and BPIE recommend that at least the following areas of interest be considered when measuring IEQ: thermal comfort, illuminance, indoor air quality, and noise. At this time, it is not common to find an IoT device that is suitable for dense deployments in schools, university campuses, hospitals, and office buildings, among others, that measures variables in all of the above areas of interest. This paper presents a solution to the problem previously outlined by proposing an IoT device that measures variables across all of the aforementioned areas of interest. Moreover, in a radio frequency network with a tree-like structure of IoT devices, this device is able to assume the roles of sensor and hub node, sensor and router node, and only sensor node. The experimental results are satisfactory, and the detailed system design ensures the replicability of the device. Furthermore, the theoretical analysis paves the way for high scalability.

## 1. Introduction

Indoor Air Quality (IAQ) has been a topic of general interest for a long time, as evidenced by the existence of regulations in different countries [1]. However, it is part of a broader concept called Indoor Environmental Quality (IEQ), which some authors consider to encapsulate IAQ (see section GreenTip 9–5 of [2]). Although some current regulatory attempts on IEQ only partially address the new concept [3], there are others that consider it comprehensively [4]. Given that standards and regulations typically appear as a result of current or future needs, it is reasonable to expect that the emerging field of IEQ will require new scientific and engineering contributions.

At present, and in relation to IAQ first and IEQ later, it can be said that the topics that have been and are being most frequently addressed in the scientific literature are the following:

- Ventilation status and contaminant detection: Indoor ventilation status is generally related to the concentration of naturally generated contaminants. This often affects the health and productivity of the compartment’s occupants [5,6,7,8,9]. In addition to the above, the detection of non-natural contaminants, introduced accidentally or intentionally [10], is also a matter of interest because they can also affect the health of living beings.
- Energy consumption: The energy consumption of Heating, Ventilation, and Air Conditioning (HVAC) systems is usually significant compared to other energy-consuming systems in buildings. As a result, this is a topic where proposals are continually being made [11,12,13,14,15,16]. In [17], a set of circuits of complementary activities is designed and successfully used to both measure variables related to IEQ and detect inappropriate energy consumption. Particularly noteworthy is the effort made to obtain low-cost devices that are also open hardware and software. The extensive literature review presented in [18] discusses how various IoT systems have been used to reduce energy consumption in buildings while increasing occupant comfort. Although the term IEQ is commonly applied to buildings for people, there are some industrial facilities, such as greenhouses, where controlling environmental variables while optimizing energy consumption is equally important [19].
- Comfort levels: According to chapters 8 of [2] and 3 of [9], the areas of interest of variables to establish IEQ conditions are as follows: thermal comfort, illuminance, indoor air quality, and noise.
  Aside from the limitations imposed by the absence of health hazards, the fact is that comfort levels with respect to various parameters of environmental interest are not the same for all individuals in all scenarios. Several researchers say that different factors that have an influence are age [20], activities prior to entering a new space [21], the metabolic rate of individuals, the insulation provided by clothing [22], and the gender of people (chapter 2.1 in [23]), among others. Therefore, in multi-user rooms, it is sometimes difficult to satisfy everyone equally. This makes measures such as Predicted Mean Vote (PMV), Actual Mean Vote (AMV), and Predicted Percentage of Dissatisfied (PPD) interesting [22].
  Another alternative to explain the complicated thermal scenarios related to comfort sensations and to facilitate decision making is the one presented in [24], which proposes the creation of a system based on artificial intelligence.
  In addition, the approach taken in [25] proposes to couple a network of sensors to the user in order to measure their state of comfort. By comparing the obtained data with the environmental conditions, a thermal comfort model is obtained.
- Technological details of the IoT sensor networks: Currently, system architectures commonly used in the Internet of Things (IoT) field are emerging as interesting alternatives for rigorous monitoring of quantities that influence the quality of environmental conditions inside buildings [26]. In addition, IoT systems generally facilitate the distribution of results among registered subscribers. The technological challenges faced by IoT systems regarding indoor environments are generally not very different from those faced by other IoT systems. Scalability is one of them. In this respect, the development proposed in [27] stands out. This reference shows the deployment of 165 sensor units throughout New Zealand to provide IAQ measurements in school classrooms.
  Another decision of great relevance is to determine which communication alternative is the most suitable for the different devices of an IoT system. In this respect, the hardware approach proposed in [28] adds flexibility to this decision. The authors provide several wired and wireless communication alternatives for each sensor unit.
  According to [29], low-cost sensors such as those presented in this paper do not require a calibration certificate. The opposite is true for professional sensors, which are typically used in the construction of high-end laboratory equipment.
  In [30], the deployment of a sensor node network with infrared communication is shown to avoid electromagnetic interference problems. This could be of interest in hospitals and other applications where there is equipment whose operation could be affected by electromagnetic radiation.
  Finally, it should be noted that when sensor nodes cannot rely on an external power source, it is necessary to use batteries or some energy harvesting strategy. Although this problem is not frequent when measuring indoor variables, since usually all buildings offer electric power, sometimes the location of the sensors may be far from any power outlet. Therefore, contributions like [31] are of interest. The authors of [31] show that they consume much more energy when using the sensors than when transmitting the information obtained. This observation could be used to optimize the time interval selected to collect information from the sensors.
- Stakeholders: From our point of view, the beneficiaries of using an IoT network to measure indoor variables are as follows:
  – The users of the space, because they can establish more rigorous indoor environmental control strategies and allow them to detect extreme scenarios that require specific actions.
  – Architects and building designers, because the feedback from low-cost sensor networks helps them improve their designs by studying environmental and behavioral data over time, improving the comfort and experience of people in buildings [32,33].
  – Decisions makers also gain benefits because they can implement their regulations with scientific rigor [34].

The main objective of this work is to present a sensor device for measuring temperature, relative humidity, CO_2_ concentration, illuminance, and noise in classrooms of a university campus. In order to get an idea of how suitable the environment is for the users of an indoor space, the measurement of these variables could be of great importance. This contributes to practically all the above-mentioned research lines. This device has the capability to be integrated into a Radio Frequency (RF) sensor network for IoT systems. In addition, with the intention of being able to operate in university campuses of different sizes, the device facilitates a sufficient scalability of the IoT sensor network due to the different roles the device can assume in the network, and the flexibility the whole sensor system offers in the way it behaves. The device can assume the roles of sensor and hub node, sensor and router node, and only sensor node.

There are several devices in the literature that measure different indoor environmental variables. Nevertheless, these devices frequently fail to perform measurements in all areas of interest for IEQ [2,9], or they are not IoT. Therefore, in the scenario of needing to measure variables in these areas and that the system is also IoT, it is necessary to combine several of the devices mentioned above. Nevertheless, this is costly due to the replication of units in several devices (e.g., power supplies, chassis, communication devices, and so on), decreases the reliability of the set, increases the power consumption, etc. The subject of this work is to propose a solution to the above mentioned drawbacks. In this context, it should be emphasized that our goal is not to use the cheapest sensors and microcontrollers available on the market, but to use the most suitable ones in terms of performance and to integrate them all on the same Printed Circuit Board (PCB).

The above was the main motivation for this work, which led to the design of an IoT sensor device. Such a device can measure variables in all areas of interest for IEQ according to ASHRAE [2] and BPIE [9]. Therefore, the contributions of this work are as follows:

1. To develop a device that measures a selected set of variables, namely: air temperature, relative humidity, illuminance, noise, and CO_2_ concentration, that may be of interest to indoor users according to [2,9].
2. To enrich the above sensor with high scalability.

This device could be useful in a number of applications, including the following: (1) IEQ scoring; (2) detecting extreme environmental scenarios in classrooms, hospitals, libraries, offices, etc.; (3) obtaining information that allows for more effective energy consumption planning in buildings; (4) verifying the occupancy status of spaces, and so on.

The remainder of this paper is organized as follows: Materials and methods are detailed in Section 2. In Section 3, results and discussion are shown. Finally, the conclusions are given in Section 4.

## 2. Materials and Methods

First, the capabilities of the sensor device to be developed were established. This is covered in Section 2.1. Section 2.2 is dedicated to explaining the choice of the communication alternative between sensors. In addition, the design and construction of the sensor node is presented in Section 2.3. Moreover, a final series of tests was conducted to verify the correct operation of the sensor node in a real-world deployment scenario, which is explained in Section 2.4.

### 2.1. Capabilities That the Sensor Node Should Have

In general, as explained in Section 1, to respond to the topics of common interest in IEQ, the capabilities that the sensor node should have are the following:

- Topic “Comfort levels”: In accordance with [2,9,35,36], it is important to measure temperature, humidity, illumination, CO_2_ concentration, and noise. It is worth mentioning that the measurement of air temperature and relative humidity allows one to obtain an estimation of thermal comfort according to [37,38,39,40,41,42,43].
- Topic “Ventilation status and contaminant detection”: Measure CO_2_ concentration because it is an indirect indicator of the degree of ventilation and undesirables air pollution levels in a room [2,35]. In addition, measurements of CO_2_ concentration are of interest in themselves due to the fact that high concentration levels can cause discomfort and headaches, among other adverse human health effects [44].
- Topic “Energy Consumption”: Enable corrective actions to be taken to improve energy consumption, based on information provided by the sensors.
- Topic “IoT network technology”:
  1. Have the capability for sensor nodes to communicate via RF until the information reaches the hub node of the RF network, as this reduces the cost of deploying the sensor network compared to wired alternatives.
  2. Be scalable enough to adequately monitor classrooms on a university campus.
- Topic “Stakeholder Support”: Sensor nodes should be able to support indoor environment stakeholders by providing measures of interest to them.

### 2.2. Selection of the Communication Alternative between Sensors

In this work, we have chosen a wireless sensor-to-sensor communication network over the wired option. This decision is supported by the following considerations:

1. Low cost of the wireless alternative compared to wired.
2. Small length of messages to be sent and low transmission frequency, which minimizes the negative impact of lower speed in RF communications compared to wired.

Among the available wireless alternatives, we chose MiWi, which is a free variant of ZigBee, developed by Microchip Technology Inc. (Chandler, AZ, USA). However, it is worth mentioning that other similar options (for example, WiFi, BLE, and others) would have achieved the same objectives. However, for the sake of convenience in this work, we chose to use the alternative suggested above.

Both ZigBee and its MiWi variant are based on the IEEE 802.15.4 standard [45], which allows the implementation of peer-to-peer and star network configurations. This standard suggests the possibility of creating more complex communication networks, such as mesh networks. However, mesh networks are not part of IEEE 802.15.4 (chapter 5.5.1 in [45]), and it is up to each implementation to provide this type of network (or other alternatives). Mesh networks introduce the complication of establishing multi-hop routes for messages.

In general, communication implementations based on IEEE 802.15.4 that support complex topologies must periodically check which nodes are active on the network and which are within range of each other. This allows routing tables to be updated to establish communication routes between all nodes. However, this reduces efficiency because network management messages must be issued in addition to device information messages.

**Figure 1:**
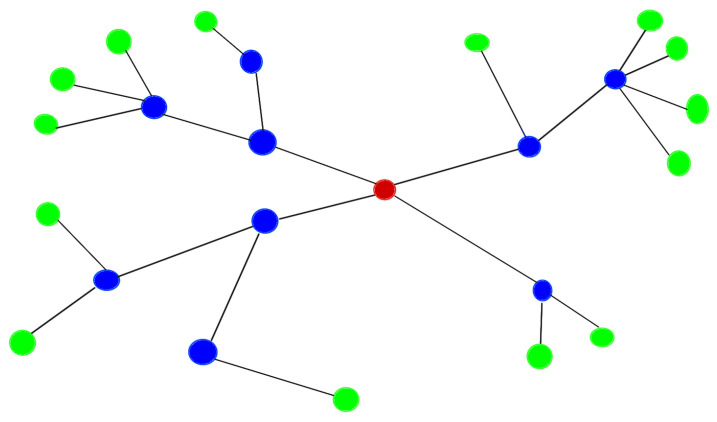
Example of a tree network. Red bubble: PAN coordinator. Blue bubbles: sensor nodes that are repeaters. Green bubbles: sensor nodes that are leaf nodes.

In this work, the networks are configured to transmit indoor environmental measurements where the number of devices is constant and their location is fixed for long periods of time. Therefore, we decided to deploy a network that does not support dynamic node incorporation to ensure that the Personal Area Network (PAN) coordinator does not have to broadcast management messages, thereby increasing network efficiency. In addition, we achieved the above by using a peer-to-peer (P2P) network that supports a static configuration with a tree network topology (see Figure 1).

In the tree network of this proposal, each node knows both to which other node it must send its messages and the messages it receives from other nodes. This is done in order for them to reach the final concentrator. In addition, the address to which it should send the message is entered when the software is compiled.

### 2.3. Sensor Node Design and Construction

The most prominent components of the system are the following:

- Microcontroller: dsPIC33EP512GM604 (https://www.microchip.com/en-us/product/dsPIC33EP512GM604, accesed on 20 May 2024).
- Communications SoC: MRF24J40ME (https://ww1.microchip.com/downloads/aemDocuments/documents/OTH/ProductDocuments/DataSheets/70005173A.pdf, accesed on 20 May 2024).
- Sensors:
  – Luminosity: VEML7700 (https://www.vishay.com/docs/84286/veml7700.pdf, accesed on 20 May 2024).
  – CO_2_ concentration: SCD30 (https://sensirion.com/products/catalog/SCD30/, accesed on 20 May 2024).
  – Temperature: SHT31 (embedded inside the device SCD30, https://sensirion.com/products/catalog/SHT31-DIS-B/, accesed on 20 May 2024).
  – Relative humidity: SHT31 (embedded inside the device SCD30, https://sensirion.com/products/catalog/SHT31-DIS-B/, accesed on 20 May 2024).
  – Microphone: MKD-1365 (https://docs.rs-online.com/f768/0900766b811685df.pdf, accesed on 20 May 2024).

**Figure 2:**
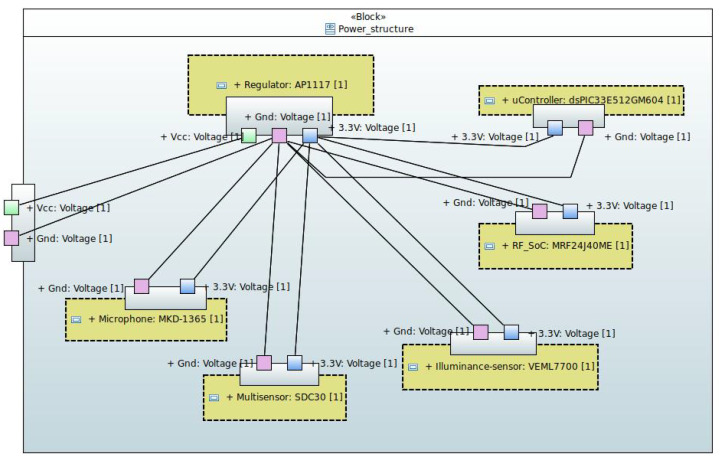
Internal block diagram of the sensing device power structure.

Some of the key data of the sensors used in the device are listed in Table 1. In addition, the internal SysML block diagram of the power supply structure of the sensor device is shown in Figure 2. Specifically, an external 5 V DC power supply is used to provide a supply voltage of 3.3 V DC for all components by using the AP1117 voltage regulator. At this point, it is worth mentioning that, in the case under study, since the maximum current consumption is low and the classroom temperature does not exceed 50 °C, it is not necessary to place a heat sink on the voltage regulator.

**Figure 3:**
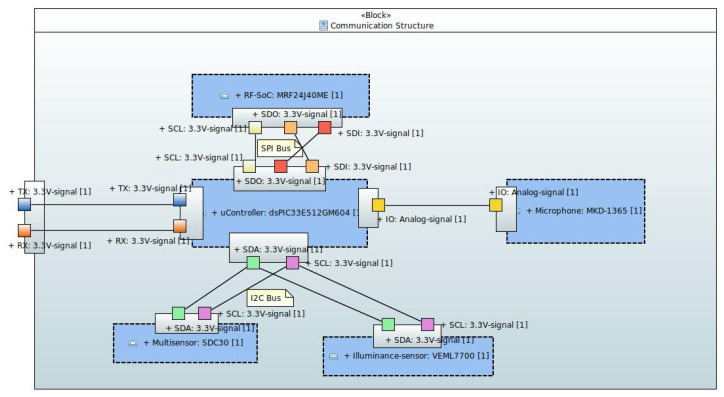
Internal block diagram of the sensig device communication structure.

Figure 3 shows the internal SysML block diagram of the communication structure and the components within the sensor device. In this figure, the temperature, humidity, CO_2_ sensors (integrated in the SCD30 device), and the luminosity sensor (VEML7700) communicate with the microcontroller via an I^2^C bus. The noise sensor (MKD-1365) conditioning circuit provides an analog signal to the microcontroller. Finally, the microcontroller communicates with the RF transmission SoC (MFR24J40MC) via an SPI bus. The board provides a USART connector as an external interface, in case the node acts as a hub. This is because the hub needs to supply the Internet entry point with both all data received from other nodes and all locally generated data. The Internet entry point is a single board computer with a USART interface that sends the information to the database server. An RF (antenna) interface is also provided to send and receive messages wirelessly.

**Figure 4:**
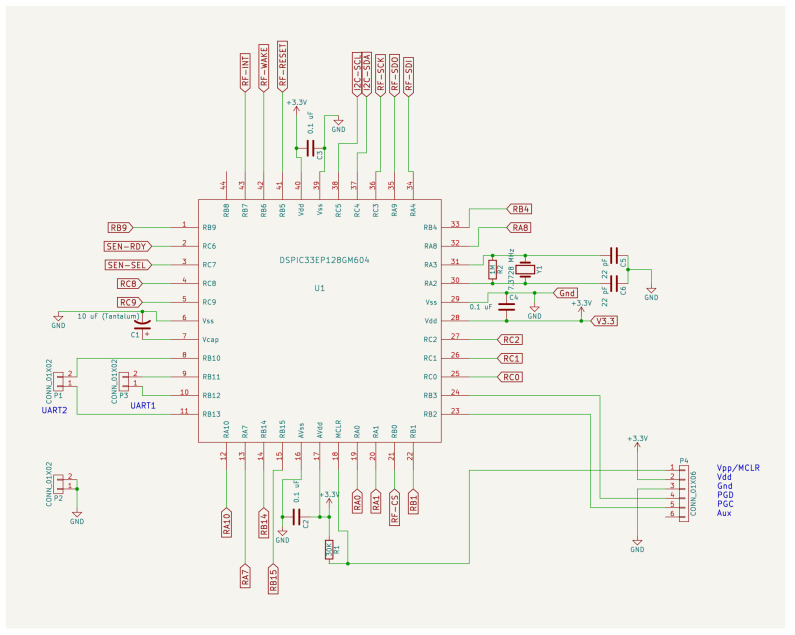
Schematic design of the electronic circuit built in this work. Microcontroller.

**Figure 5:**
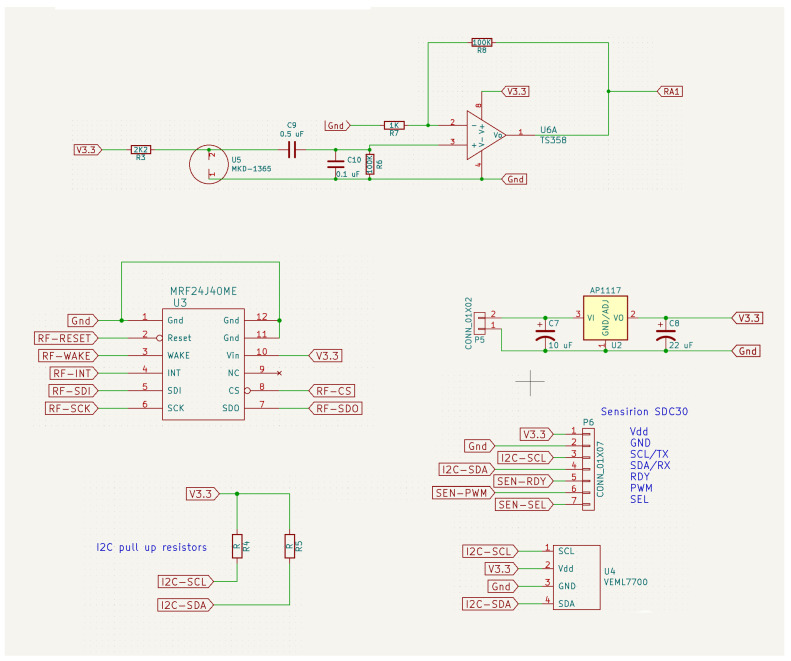
Schematic design of the electronic circuit built in this work. Other components than microcontroller.

Figure 4 and Figure 5 contains the electronic schematic design of the developed device. The microcontroller is shown in Figure 4. The microphone signal conditioning circuit is shown in the upper part of Figure 5. The RF communication SoC (MRF24J40ME) is shown on the left in the middle of the figure. To the right of the center of the figure is the voltage regulator. Finally, the I^2^C interface devices (SCD30 and VEML7700) can be seen at the bottom.

**Figure 6:**
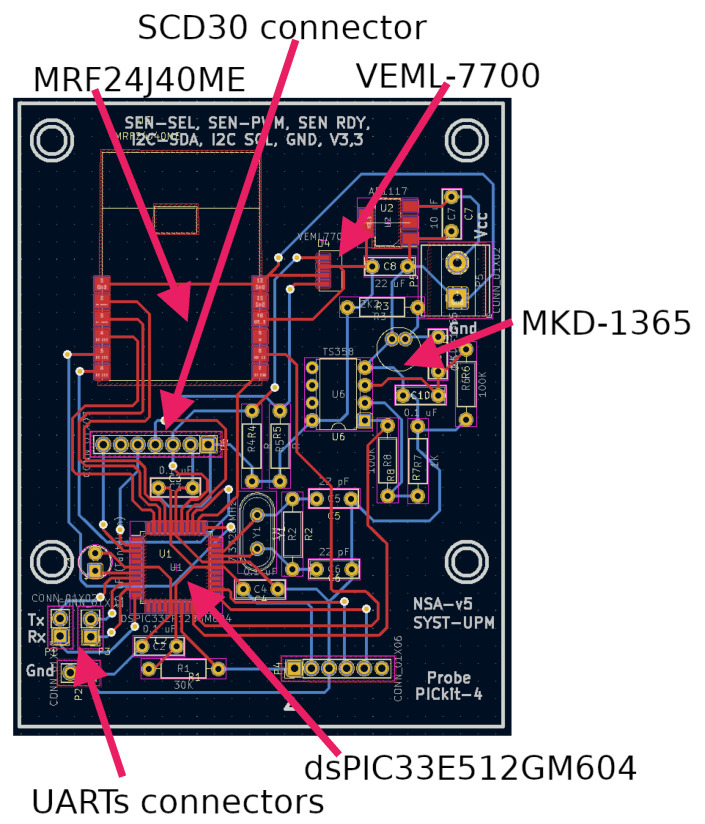
Design of the PCB of the electronic device, pointing out its most outstanding components.

**Figure 7:**
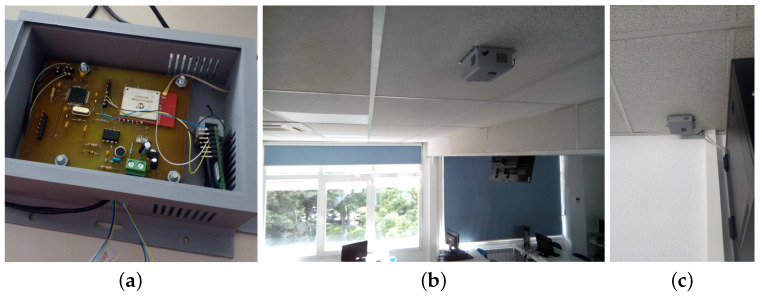
(**a**) Sensor node box. (**b**) Sensor node placed on the ceiling in the middle of the classroom. (**c**) Sensor node placed at the back of the classroom.

**Figure 8:**
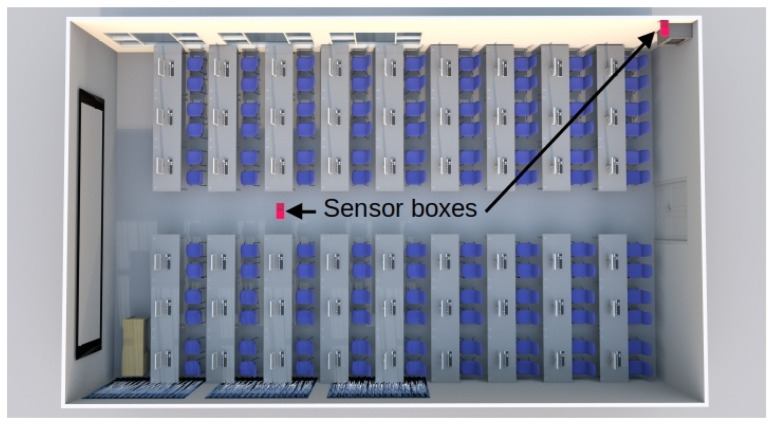
Top view of the classroom with sensor box locations.

**Figure 9:**
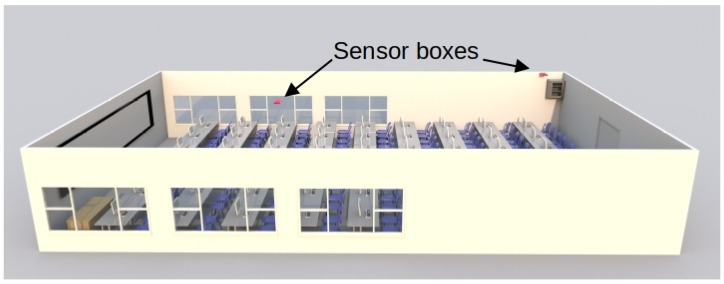
Left side view of the classroom with sensor box locations.

Figure 6 shows the Printed Circuit Board (PCB) design of the electronic device. In addition, Figure 7 shows the prototype of this circuit in the classroom, and Figure 7a shows the circuit inside its enclosure. The location of the two sensor boxes (see Figure 7b,c) is firstly due to the fact that a representative point was sought where there will always be students, regardless of the number of attendees in each class. This point is located in the first third part of the classroom (see Figure 7b, Figure 8, and Figure 9). On the other hand, in order to have more information and reduce the excess of wiring in the classroom, the concentrator node was placed near the communication cabinet, as this node is also a sensor box (see Figure 7c, Figure 8, and Figure 9). The Internet switches are located in the communications cabinet, which is responsible for providing Internet access to all computers in the classroom.

### 2.4. Test Bed

**Figure 10:**
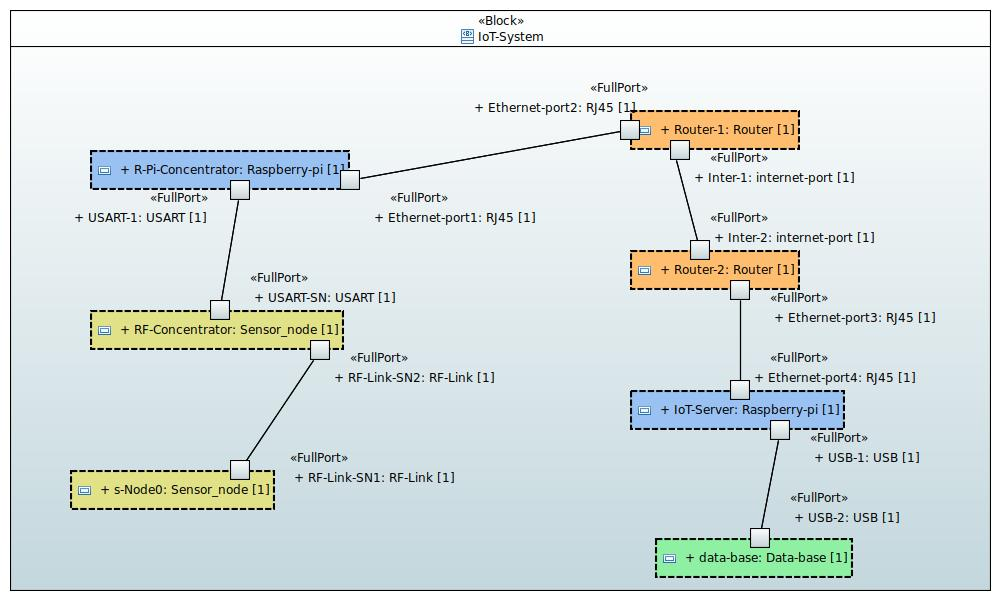
Internal block diagram of the IoT system configuration.

To test the correct behavior of the sensor node in a real-world deployment scenario, all the components needed to build a basic IoT system were created (data transfer scenarios to IoT system consumers have not been activated in the tests, as it is not essential to evaluate the behavior of the developed IoT devices). Schematically, the system responds to the component and interface design shown in Figure 10 and detailed below:

1. Sensor nodes. These are devices developed to collect indoor environmental measurements and transmit them via RF to a concentrator node.
2. Concentrator node. It is a sensor node in which, in addition to generating the information acquired at its location, it collects the information received from the sensors deployed in the same RF network. The collected information is sent, through a USART channel, to an element with an Internet connection (injector node).
3. Injector node (Internet entry point). It is responsible for receiving information from the concentrator node and injecting it into the Internet to a database server, where the information will be permanently stored.
4. Message router node (Internet router). The injector nodes of an installation connect to a router, which knows how to redirect messages to reach their destination.
5. Database server node (Internet endpoint). It is the node in charge of receiving the information from the Internet and storing it permanently in a database.

The schematic code that is executed in the PAN coordinator of the network is shown in Algorithm 1. In addition, the code that is executed in each repeater node or leaf node is shown in Algorithm 2. It can be seen that the most notable difference is that the latter nodes do not raise the network, but are incorporated into the network previously established by the PAN coordinator.

On the other hand, the data to be transmitted or received from other nodes are sent by RF using the MiWi protocol.
**Algorithm 1** Schematic code design of PAN coordinator.**Init** system clock, peripherals, and sensors;**Initialize** MiWi protocol and raise the RF network by selecting the least saturated channel;**LOOP**  **PARALLEL ACTIVITIES**     **each 5 min**       - Measure air temperature, relative humidity,       illuminance, and CO_2_ concentration       - Prepare to send all measurements by USART,       including noise and reset maximum noise to zero     **each 100 μs**       - Get a noise measurement       - Save the noise measurement in       maximum noise if it is higher than       the current maximum noise value     **continuously**       - if a message from other node is received,       prepare to retransmit it by USART     **continuously**       - Send by USART prepared messages  **END PARALLEL ACTIVITIES****END LOOP**

**Algorithm 2** Schematic code design of a full future device.
**Init** system clock, peripherals, and sensors;**Initialize** MiWi protocol and look for the channel which operates the PAN coordinator;
**LOOP**
  **PARALLEL ACTIVITIES**     **each 5 min**       - Measure air temperature, relative humidity,       illuminance, and CO_2_ concentration       - Transmit all measurements by RF       to the following net device including noise       and reset maximum noise to zero     **each 100 μs**       - Get a noise measurement       - Save the noise measurement in       maximum noise if it is higher than       the current maximum noise value     **continuously**       - if a message from the other node is received,       retransmit it by RF to the following net device  **END PARALLEL ACTIVITIES**
**END LOOP**



## 3. Results and Discussion

### 3.1. IoT System Testing

As a preliminary testing approach, five IoT system test groups were established:

1. Initialization of the microcontroller and its peripherals.
2. Verification of sensors operation.
3. Verification of RF communication between sensor nodes.
4. Verification of the communication between the concentrator and the injector node.
5. Verification of the communication between the injector node and the database server.

The previous tests were of individual components and integration. The defects found were minor software defects, and no design or specification defects were found. Therefore, we moved on to deploying the IoT system for long-term testing. The test deployment used two sensor nodes installed in a university classroom (see Figure 7b,c). In addition, one of the nodes was configured as a PAN coordinator and deployed at the end of the classroom (see Figure 7c). This sensor node was connected via a USART to a Raspberry-pi Single-Board Computer (SBC), which acted as an injector node. This node was responsible for sending messages to the database server using an open port on a switch near its location.

On the roof, in the first third part of the classroom (see Figure 7b), a sensor node was placed in charge of communicating with the concentrator node via MiWi. In addition, a database server node was placed in a building near the classroom. This node was also housed in another SBC (Raspberry-pi) with an external hard disk connected through a USB channel.

The variables that were considered in our sensor nodes usually evolve slowly, except for noise. Therefore, it can be considered reasonable to take measurements every 5 min, without the risk of losing significant information. However, sound can have considerable variations in the same interval. Therefore, we decided to sample this signal with much smaller periods.

With respect to the sound signal, the sampling period was 100 μs, and we kept the maximum of the samples taken during a 5 min interval. Once this time has elapsed, the maximum of the samples taken from the sound signal is transmitted together with the data obtained from the other sensors. Then, all the variables are reset and the process is repeated indefinitely (see Algorithm 2).

### 3.2. Scalability of the IoT System

The previously mentioned does not make sense if the correct operation of a reasonable set of sensor nodes per hub node cannot be guaranteed. Therefore, we have considered an upper limit, not mandatory, of 120 sensor nodes per hub node. The reason why we chose the number 120 is that we are considering an extreme scenario, where we could find ourselves in the case of 120 classrooms in a building. However, we explain later in this paper how the system built can serve an extraordinarily large number of classrooms.

With 120 sensor nodes, the hub node needs to spend in RF communications less than 0.1% of its period of 5 min to take measurements of its own sensors (MRF24J40 data rate = 250 kb/s, data frame length = 53 bytes (of which 30 bytes are payload), which means that it takes 0.20352 s to receive the information from the 120 sensor nodes).

Assuming a USART configured conservatively at 9600 baud with one start bit and two stop bits per byte, it takes 4.43666667 s to transmit the information from all the sensors ((30 data bytes + 2 synchronization bytes) × 121 sensor nodes, including the hub node) to the Internet access point. Total USART time plus radio frequency = 4.640187 s. Therefore, the hub node spends 1.5% of 5 min to provide communication service.

Furthermore, the time spent by the hub node in transmitting messages to the injector node from other network nodes is less than 0.2% of the transmission period of its own data (i.e., the hub node’s data), which is 5 min. This occurs when the USART baud rate is equal to 115,200 baud.

One of the most critical parameters for good scalability of the sensor segment of the IoT system is the number of transactions per minute supported by the database system. In this work, to intentionally reduce behavioral expectations, we have used a modest but sufficient non-distributed database (SQLite). For non-distributed databases, the number of transactions per minute is highly dependent on the disk technology used. Additionally, considering the type of transactions when storing new data, which is equal to 32 bytes in length per transaction in this first deployment, the number of transactions per minute can vary from 350 for a very low-end external HDD to more than 60,000 for a high-end external SSD.

In this work, the high-end SSD option allows up to 495 hub nodes with 120 sensor nodes for each hub. That is, by distributing two sensor nodes per classroom, 495 × 120/2 = 29,700 classrooms could be monitored (Under these conditions, a disk of only one terabyte would take almost a year to overflow (without taking into account file system management data). $10^{12}$/(59,895 ×12×24×5×32)=362.32 days. 495 × 121 = 59,895 sensor nodes, 12 communications per hour, 24 h per day, 5 database transactions per communication, 32 bytes on disk per transaction). Obviously, this figure does not take into account a number of realistic factors that could significantly reduce the number of classroom that could be monitored. Some of these factors are as follow: (1) need to resend messages due to collisions with other devices in the same network, (2) interferences with other networks in the ISM-Band (i.e., WiFi, ZigBee, Bluetooth, etc.), (3) attenuation due to poor positioning of devices with respect to concrete walls or thick brick walls in combination with lack of repeater nodes, and so on.

Regarding the need to resend messages due to collisions caused by multiple devices sending messages simultaneously, once the first collision is detected, the MiWi and ZigBee protocols implement a contention mechanism to reduce the probability of future collisions. However, the proposal presented in this work does not have this problem inherently. In short, the above depends on the proximity in which the transmitter nodes are located in each specific deployment, taking into account that a transmitter can be a leaf node or a repeater node. Furthermore, since the constructed sensor nodes have a periodic actuation mode, there is no collision if the nodes within the radio frequency range coordinate to phase shift their actuation instants. In experiments conducted in the early stages of this work, eight test sensor nodes in their preliminary version were placed in the same classroom and no collisions were detected. This was achieved by following the procedure of not starting any pair of sensor nodes at the same time. Specifically, if the start-up delay of a node with respect to the time at which the previous node was started is greater than the time it takes to transmit a message, and also all nodes are started within a time interval shorter than the period at which they transmit, then there are no collisions in the communication between sensor nodes.

With respect to interference with other networks in the ISM band, the most prominent protocols (WiFi, Bluetooth, and ZigBee, among others) implement channel hopping. In this work, experiments were conducted for the case under study to determine the need to use channel hopping. However, the result was that it was not necessary. Therefore, only the least noisy channel was sought for the PAN coordinator to start the communication network on that channel. Naturally, in a dense network cohabitation scenario with long channel occupancy intervals (e.g., streaming video, streaming audio, transmitting large amounts of data, etc.), channel hopping becomes an essential alternative.

Finally, regarding the attenuation of the signal by the walls, it is true that the transmitted signals can suffer great attenuation due to the susceptibility to electromagnetic radiation of the type of material used in the construction of the buildings. In fact, concrete materials are the ones with the highest absorption to electromagnetic radiation [46,47]. In the case under study, being aware that all this may affect the signal transmission [48], we propose to use repeaters where necessary. To this end, in this work we have built a protocol for networks with a static tree configuration based on peer-to-peer networks. This alternative virtually eliminates network maintenance messages while allowing an unlimited number of repeater nodes in any possible branch of the tree. The only notable limitation of this proposal, as with the other communication alternatives, is the saturation that could be generated by including a very large number of sensor nodes in a branch of the communication network. In preliminary tests conducted at the beginning of the work, nodes capable of transmitting up to 1 km between sensor nodes in line of sight were used. However, when thick (0.3 m wide) solid brick walls were placed between the sensor nodes, the range was reduced to just over tens of meters. This problem justified the importance of being able to deploy as many repeater nodes as needed in any given scenario, which is one of the main pillars of this work.

However, the margin between the calculated figure and the number of classrooms that a university campus actually has is so large that we believe the proposal is still effective. Moreover, the architectural design proposed in this work allows for even greater scalability. For example, there is nothing stopping us from proposing more database server nodes to serve different groups of nodes.

**Figure 11:**
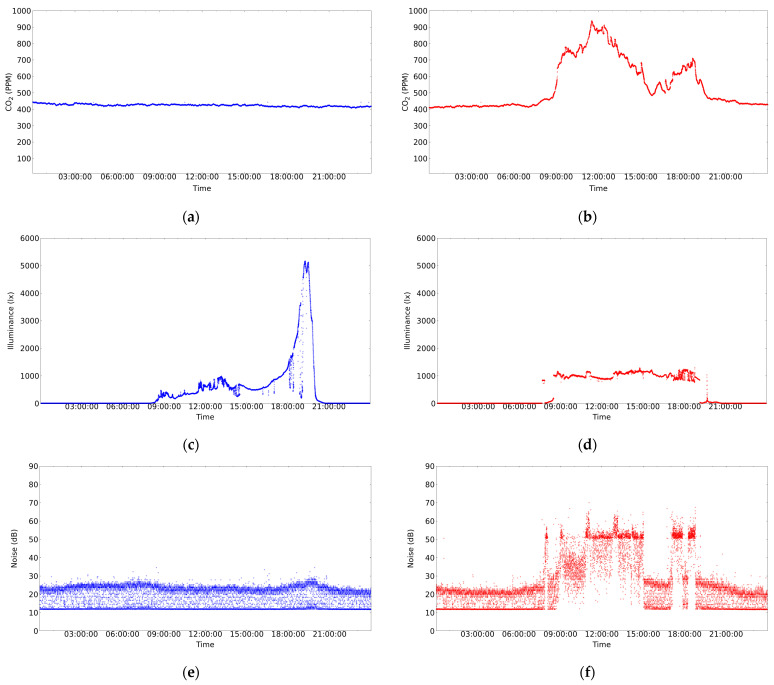
Measurements taken during a weekend day (on Saturday): (**a**,**c**,**e**). Measurements taken during a week day (on Wednesday): (**b**,**d**,**f**).

In any case, and with the intention of performing a moderate stress test, in this work two sensor nodes were deployed using a period of 5 s (recall that the system is designed to work with a 5 min interval, see Section 3.1). This was done with the aim of generating a stress on the database server similar to that generated by 120 sensor nodes transmitting every 5 min. In addition, a SEAGATE 1TEAP5-500 HDD was used in the database server. The aforementioned system has been operating satisfactorily for several months without interruption. Figure 11 shows the results of the measurements taken during a week day (Wednesday) and a weekend day (Saturday) in the classroom under test. The maximum capacity of the classroom is 108 students. However, as can be seen in Table 2, the classroom is not always full because not all subjects have the same number of students enrolled. In addition, there are no classes on Monday mornings and Friday afternoons. Therefore, only the cleaning and maintenance staff enters the classroom during the periods when there are no classes.

### 3.3. Measurement Results

The results of some of the measurements carried out are shown in Figure 11. As can be appreciated, the variables of interest were as follows: CO_2_ concentration, luminosity, and noise during a day with no activity on the university campus (see Figure 11a,c,e), and a day with activity on the university campus (see Figure 11b,d,f). Briefly, the results shown in Figure 11b,d,f represent the evolution of the variables measured in the classroom used in the experiment, where lessons were held from 9:00 to 15:00 and from 17:00 to 19:00.

The graphs in Figure 11 show some details that deserve to be commented on:

1. In Figure 11b, a very significant variation of the CO_2_ concentration can be observed with respect to Figure 11a. This is due to the fact that in the first case the classroom is occupied and in the second case the classroom is empty. Figure 11b shows that from 00:00 to 08:00 the classroom is empty and, logically, the windows are closed. However, at 08:00, the cleaning assistants enter the classroom and perform the morning task of preparing the classroom. This activity usually takes between 10 and 20 min and is carried out by one to three people. Once the cleaning is done, the classroom is open for students to enter and by 09:00 most students are in the classroom. Note that this is reflected in a significant change in the CO_2_ concentration of the classroom that occurs in a short interval of time. The class then runs normally for about 110 min. After this time, there is a short break due to the change of subject in the classroom. In addition, it seems that more students attend the next part of the class because the level of CO_2_ concentration reached is higher than in the previous case. However, between 12:00 and 13:00, the students probably felt uncomfortable with the environmental conditions and may have opened the classroom windows. This must have caused the CO_2_ concentration to drop, as can be seen in Figure 11b. After that, there was no class from 15:00 to 17:00, although some students may have stayed in the classroom to work. Then, from 17:00 to 19:00, students returned to the classroom. At this point, it is worth mentioning that it is likely that the students came to class gradually because many of them work and it is difficult for them to arrive in time for the start of classes in the afternoon.
  Finally, at 19:00, there was no class that day, and it was observed that the students left the classroom and the CO_2_ concentration dropped rapidly. This rapid decrease in CO_2_ concentration may be due to a janitor entering the classroom and opening the windows. As shown in Figure 11b, after 19:00, the CO_2_ concentration decreases until it reaches the support level in the Earth’s atmosphere (just over 423 PPM). This level is maintained until 00:00, at which time the next day’s data begins to be recorded.
2. Since CO_2_ is an indicator of the degree of ventilation and pollution level of an indoor space (see Section 2.1), the correlation between this indicator and the number of students in the classroom should be significant. In this paper, from the data shown in Figure 11b, it is experimentally demonstrated that the above correlation is 0.7709802 with *p*-value = 5.517182 × 10 ^−58^.
3. Figure 11c shows a change in luminosity from 8:00, which increases almost exponentially from 17:00 and drops sharply at 20:00. This is due to the fact that the classroom has windows on its side walls (see Figure 7b), and these have an east–west orientation. In Figure 11c, there are no brightness peaks in the morning, because there are large trees located on the east side of the classroom that filter the light until the sun rises above the lintel of the windows. However, in the afternoon there is an interval of just under two hours in which the sun can enter unobstructed on the west side before setting. On the other hand, Figure 11d shows an almost uniform behavior, because artificial lighting is used practically all day and the sunshades on the west side of the classroom are closed. In summary, Figure 11c shows a luminosity peak around 20:00 on a day when there is no activity in the center. No one is in the classrooms, the sunshades are open, and the sun shines directly into the classroom without any filter and then quickly disappears behind the horizon. On the other hand, on a school day, the students lower the sunshades and turn on the artificial light for the whole day (see Figure 11d).
4. Finally, in relation to noise, a relatively uniform behavior can be observed during any given day of the weekend (see Figure 11e). However, it should be clarified that absolute silence is not perceived in the classroom. This is due to the sporadic activity of small wild animals in the garden on the east side of the classroom (such as woodpeckers, blackbirds, and magpies, among others). To this must be added the constantly operating equipment located in the classroom (see communications cabinet to which the classroom computers are connected in Figure 7c).
  Figure 11f shows the measurement of the sound level in the classroom during a school day. It can be observed that from 0:00 to about 8:00 the recorded noise level is similar to that of a day without classes (e.g., see Figure 11e). At 8:00 a significant peak is detected that lasts for about ten minutes. This corresponds to the activity of the cleaning staff in the classroom. Between 8:00 and 9:00, the noise gradually increases, which could be due to students gradually entering the classroom. At 9:00 there is a peak above 50 dB, which marks the beginning of the first class of the morning, and between 9:00 and 11:00 the noise in the classroom is higher than during the night, with a maximum level around values slightly above 50 dB.
  Shortly before 11:00, the classroom noise rises for just over 10 min and exceeds 65 dB. This event appears to coincide with a class change and is consistent with a significant number of students remaining in the classroom talking. This is followed by the next class from 11:00 to 13:00. During this time, there seems to be more collaboration in the classroom as there is a higher density of sound samples with values slightly above 50 dB. Again, at the end of the class at 13:00 there is a peak similar to the previous class change, above 60 dB. There is another class from 13:00 to 15:00, and at the end of this class the noise level returns to the base level with a maximum noise value around 25 dB to 30 dB.
  In the afternoon, there is a class from 17:00 to 19:00, and in the middle of the class there is a break of about 20 min around 18:00. During this break, it seems that most of the students leave the classroom because the noise level drops to the baseline profile, with a ceiling between 25 dB and 30 dB. The class then resumes and ends at 19:00, at which time an abrupt drop in noise is detected. In addition, between 19:00 and 21:30, the noise level gradually decreases as the university center empties of students and workers. Finally, after 21:30, the behavior is similar to that of a day without classes (e.g., see Figure 11e).

In relation to the graphs presented in this section, it is pertinent to mention that the sensor nodes also measure temperature and relative humidity. However, these measurements have not been presented in this paper because they do not reflect significant changes in these variables between working and non-working days. We believe that this is due to the insulation of the walls and the fact that the measurements were taken on days with very good environmental conditions in spring.

### 3.4. Discussion on the Contribution of This Work Based on the Type of Sensors Used in Other Devices Designed to Measure Indoor Enviromental Variables

In this section, a comparative analysis is made between some researchers published in the scientific literature and the work presented here.

First of all, it is worth noting that the works found in the scientific literature on devices for measuring indoor environmental variables are generally very good. However, in some cases, the given solution involves deploying multiple devices with different sensors, duplicating radio frequency transmission systems, which increases the probability of radio frequency communication collisions. In addition, this problem is exacerbated by the increased number of power supplies, device packaging, and unwanted higher power consumption, among other drawbacks. This complicates the system architecture and data collection and is likely to be more costly than a concentrated solution.

In relation to the above, it is also common to find IEQ measurement devices that do not include all the recommended basic IEQ information items according to [2,9] (i.e., thermal comfort, illuminance, air quality, and noise). This is probably due to the fact that their research objectives do not require this consideration. In this sense, it is very common not to pay attention to the sound level. Table 3 shows some of the works already referenced in this paper, indicating which measures they perform.

It is observed that temperature and relative humidity are measured in all experiments. The most measured variable is CO_2_ concentration, followed by illuminance and finally sound level. In addition, in some cases other variables are also measured, such as VOC, PM_2.5_, etc. An outstanding development is the one presented in [49], where, as in our paper, all IEQ areas of interest recommended for measurements are considered. However, the device presented in [49] is not IoT. Therefore, it does not have the capacity to be integrated into a network of sensors with which it works cooperatively.

The analysis presented in this section indicates a necessity for solutions that are more tailored to the specific issue of measuring indoor environmental variables and making the best use of existing communication technologies.

This paper presents a device that measures a set of different indoor environmental variables with all sensors integrated in one electronic circuit, which also covers all the measurement areas of interest for IEQ recommended by [2,9]. In addition, the device has the potential to be integrated into an IoT network. This represents a significant advantage in terms of cost-effective monitoring of a substantial number of spaces within buildings.

In summary, we recommend the design of electronic circuits that allow measurements to be made in a single device in areas of interest for the study of indoor environments and human comfort in buildings. Furthermore, we recommend that this device should be able to be integrated into an IoT sensor network. In essence, this is important because IoT allows having as many measurements as possible in a field of interest for the benefit of the population, reducing costs, providing continuous information in real-time, and facilitating decision-making, among other advantages.

## 4. Conclusions

This work presented an IoT device designed to measure indoor temperature, relative humidity, illumination, CO_2_ concentration, and noise. This device could be beneficial in applications where it is necessary to study the indoor environment. In addition, the device can be used to detect adverse scenarios in prominent spaces, such as classrooms and hospitals. Moreover, the measurements obtained can be utilized in the planning of more efficient energy usage, among other applications.

The device is equipped with a transmitter that supports communication according to the IEEE 802.15.4 standard and has enough power to reach one kilometer when the transmitter and receiver are in sight. The protocol used in the sensor nodes was MiWi, an open-source variant of ZigBee developed by Microchip Technology Inc.

This sensor node can be configured to have all available capabilities of the communication protocol (Full Future Devices). This allows it to act as a repeater node when needed, making it possible to deploy networks where not all devices are within range of the hub node.

In order to reduce the number of network management messages that could be transmitted, the mesh network configuration (a very suitable configuration for our needs, provided by ZigBee and MiWi) was abandoned in this work. However, by working on a peer-to-peer configuration, we have introduced the static information elements necessary to implement networks with a tree structure (see Figure 1). The peer-to-peer configuration is the simplest of those supported by the IEEE 802.15.4 protocol, usually requiring a very small number of network management messages. Furthermore, from our point of view, tree networks are the most suitable alternative to achieve the objectives to be considered in a deployment of IoT devices aimed at measuring indoor environmental variables in classrooms on a university campus. The tests performed in this research demonstrate the high scalability of the proposed system, which theoretically allows serving up to 29,700 classrooms of a university campus with a single database server implemented on a single board computer (Raspberry-pi).

As future work, we plan to measure ambient light color so that the instructor can change it according to the interest of each activity.

## Figures and Tables

**Table 1:** Some relevant data about the sensors used, provided by their manufacturers.

Device	Accuracy	Measurement Range	Other Specifications
VEML7700	N/A	0–167,000 lx	Ambient Light
(Vishay Intertechnology			Resolution: 0.005 lx
Malvern, PA, USA)			I^2^C Bus Voltage
			Range: 1.7 V to 3.6 V
SCD30 CO_2_	±(30 ppm + 3%)	0–40,000 ppm	Repeatability:
(Sensirion AG			10 ppm
Stäfa, Switzerland)			Temperature
			stability: 2.5 ppm/°C
SHT31 Temperature	±0.5 °C	−40–120 °C	Repeatability:
(Sensirion AG			0.1 °C
Stäfa, Switzerland)			
SHT31 Humidity	±2% RH	0% RH–100% RH	Repeatability:
(Sensirion AG			0.1% RH
Stäfa, Switzerland)			
MKD1365	N/A	N/A	Sensitivity:
(Kingstate Electronics Corp.			−40 ± 3 dB
Taipei Hsien, Taiwan)			Signal to noise
			ration: 58 dBA
			Output impedance:
			2.2 KΩ
			Frequency:
			20–20,000 Hz
			Directivity:
			Omnidirectional

N/A stands for not applicable.

**Table 2:** Class schedules with the number of students in each time slot.

Time	Monday	Tuesday	Wednesday	Thursday	Friday	Saturday	Sunday
9:00–11:00		102 (DB ^1^)	102 (DB ^1^)	103 (AL ^2^)	92 (CA ^3^)		
11:00–13:00		103 (AL ^2^)	96 (OOP ^4^)	92 (CA ^3^)	96 (OOP ^4^)		
13:00–15:00			107 (AI ^5^)		102 (DB ^1^)		
15:00–17:00		102 (AL ^2^)		101 (DB ^1^)			
17:00–19:00	95 (OOP ^4^)	101 (DB ^1^)	95 (OOP ^4^)	91 (CA ^3^)			
19:00–21:00	101 (DB ^1^)	91 (CA ^3^)	107 (AI ^5^)	102 (AL ^2^)			

^1^ Subject: Data Base; ^2^ Subject: Algorithm Complexity; ^3^ Subject: Computer Architecture; ^4^ Subject: Object Oriented Programming; ^5^ Subject: Artificial Intelligence.

**Table 3:** Sensors used in related research works referenced in this article.

Reference	Temperature	Relative Humidity	Illuminance Concentration	CO_2_ Level	Sound	Others
Ali et al. [17]	×	×	×	×		×
Aparicio-Ruiz et al. [20]	×	×	×			×
Mishra et al. [21]	×	×		×		×
Kuncoro et al. [23]	×	×				
Sahoh et al. [24]	×	×				×
Wei et al. [27]	×	×		×		×
Zhao et al. [28]	×	×				×
Salomone et al. [29]	×	×	×	×		×
Phan et al. [30]	×	×		×		×
Folea and Mois [31]	×	×	×	×		×
Tiele et al. [49]	×	×	×	×	×	

## Data Availability

All data are contained within the article.
